# Shape memory and self-healing in a molecular crystal with inverse temperature symmetry breaking†

**DOI:** 10.1039/d3sc06800e

**Published:** 2024-03-08

**Authors:** Jiantao Meng, Yuan Su, Hang Zhu, Ting Cai

**Affiliations:** a Department of Pharmaceutics, School of Pharmacy, China Pharmaceutical University Nanjing 211198 People's Republic of China tcai@cpu.edu.cn; b Department of Pharmaceutical Engineering, School of Engineering, China Pharmaceutical University Nanjing 211198 People's Republic of China

## Abstract

Mechanically responsive molecular crystals have attracted increasing attention for their potential as actuators, sensors, and switches. However, their inherent structural rigidity usually makes them vulnerable to external stimuli, limiting their usage in many applications. Here, we present the mechanically compliant single crystals of penciclovir, a first-line antiviral drug, achieved through an unconventional ferroelastic transformation with inverse temperature symmetry breaking. These crystals display a diverse set of self-restorative behaviors well above room temperature (385 K), including ferroelasticity, superelasticity, and shape memory effects, suggesting their promising applications in high-temperature settings. Crystallographic analysis reveals that cooperative molecular displacement within the layered crystal structure is responsible for these unique properties. Most importantly, these ferroelastic crystals manifest a polymer-like self-healing behavior even after severe cracking induced by thermal or mechanical stresses. These findings suggest the potential for similar memory and restorative effects in other molecular crystals featuring layered structures and provide valuable insights for leveraging organic molecules in the development of high-performance, ultra-flexible molecular crystalline materials with promising applications.

## Abstract 2

Mechanically responsive molecular crystals of the pharmaceutical compound penciclovir have been reported, demonstrating ferroelasticity, superelasticity, shape memory, and self-healing, all attributed to cooperative molecular displacement.

## Graphical abstract

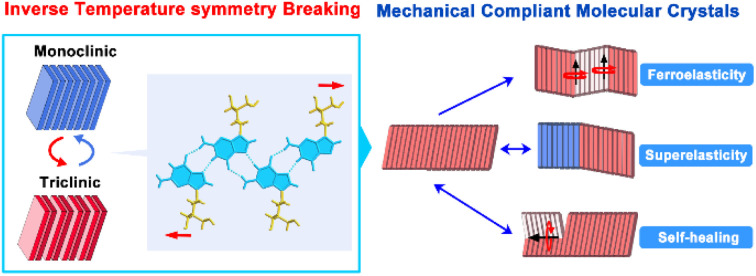

## Introduction

Polymorphic transitions, characterized by cooperative molecular motions, have gained increasing attention in the research community due to their rapidity, reversibility, and controllability.^1–4^ These transitions, often referred to as martensitic transformations in metallurgy, confer crystalline materials with structural adaptability under external stimuli, enabling a diverse range of stimuli-responsive behaviors^5^ such as the thermosalient effect,^6–8^ elastic and super-elastic deformation,^9,10^ and shape memory effect.^11–14^ When accompanied by a change in the point-group symmetry, such transitions are called ferroic phase transitions, leading to low-symmetry polymorphs that exhibit ferroelasticity, where the spontaneous strain can be reversed by applying an external stress field.^15^ Ferroelastic materials hold great promise for applications in mechanical switches, energy conversion, and information storage based on their unique properties.^15^ Recent years have witnessed the discovery of an increasing number of ferroelastic molecular crystals, which provide the advantages of low weight, low cost, easy processability, and nontoxicity.^16,17^ However, their ferroelastic behaviour has primarily been observed at room temperature because crystal symmetry generally rises as temperature increases during the ferroelastic phase transition, thereby resulting in the loss of ferroelasticity at elevated temperatures.^18^ Recent studies have unveiled an intriguing inverse temperature symmetry breaking (ITSB) phase transition in inorganic and hybrid materials, where the high-temperature (HT) form exhibits a lower symmetry than the low-temperature (LT) form, offering a promising pathway for the development of high-temperature ferroelastic materials.^19–25^ Despite these promising developments, the exploration of ITSB in molecular crystals remains underexplored.

Mechanically responsive materials, possessing mechanical compliance, have captured particular interest for their potential utilization in fields that range from aerospace materials and optoelectronic devices to artificial muscles and soft robotics.^26^ Traditionally, crystalline materials were perceived as rigid and brittle; however, recent advancements in solid-state chemistry have changed these beliefs. Over the last two decades, there has been a significant increase in the study of molecular crystals, which exhibit extraordinary flexibility.^27–31^ Recently, the shape memory effect, which enables materials to regain their original shape and functionality even after undergoing significant deformation, have been extended to molecular crystals.^32–34^ Research reveals that these molecular crystals, despite sharing similarities with shape memory alloys whose recoverability is executed by thermal-induced phase transitions, exhibit more intricate mechanisms due to their weak and complex intermolecular interactions.^11,35^ Moreover, Naumov *et al.* opened up the avenue of self-healing molecular crystals in 2016.^36^ Since then, a handful of cases have been reported, and most were discovered during phase transitions.^9,37–42^ However, the mechanisms underlying self-healing in crystalline materials remain somewhat ambiguous. To date, it remains a great challenge to discover and design new mechanically compliant molecular crystals.

**Fig. 1:**
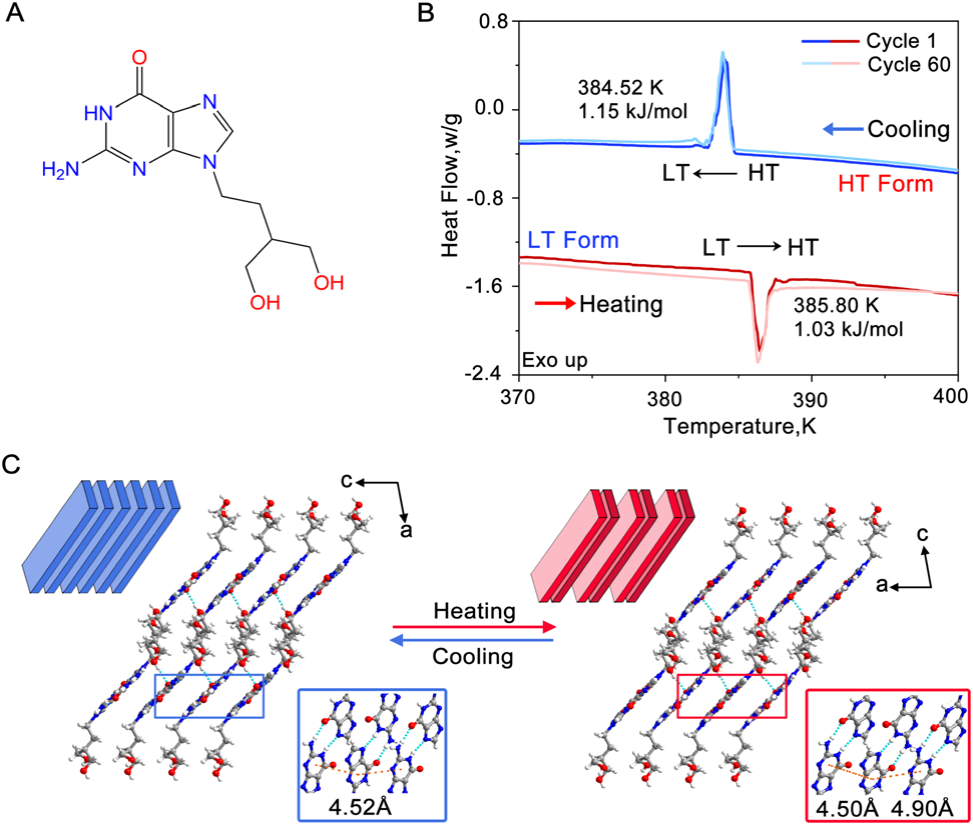
Polymorphs and phase transition of the PCV crystals. (A) Chemical structure of PCV. (B) DSC profile recorded by heating and cooling of the PCV crystals over the temperature of phase transition. (C) Layered structures of the two forms are shown together with centroid-to-centroid distances between the purine rings from adjacent layers for either structure. The hydrogen bonds are shown as blue dotted lines.

Here, we present the discovery of a mechanically compliant single crystals of a first-line anti-herpes drug, penciclovir (PCV, Fig. 1A).^43^ PCV crystals undergo an anomalous inverse temperature symmetry breaking (ITSB) phase transition, triggered by molecular motion within their layered crystal structure. This unique transition endows them with a high degree of mechanical flexibility well above room temperature (>385 K), resulting in a diverse array of self-restorative effects, including ferroelasticity, superelasticity, and shape memory effects. These crystals exhibit excellent durability, remaining robust after undergoing dozens of phase transitions. Most significantly, the structural integrity of PCV crystals can be almost entirely restored, even after severe cracking induced by thermal or mechanical stresses, attributed to the facile reformation of interlayer hydrogen bonds. This work introduces a molecular single crystal that displays the most diverse range of self-restorative effects reported to date, suggesting the potential utilization of organic molecules in the development of high-performance, exceptionally flexible, and durable molecular crystalline materials.

## Results and discussion

Colourless single crystals of PCV, exhibiting either plate-like or rod-like morphology, were crystallized from a mixed alcohol solution using a solvent evaporation method (Fig. S1†). The phase transition behaviour of these single crystals was monitored using differential scanning calorimetry (DSC) (Fig. 1B). Upon heating, the crystals underwent a first-order endothermic phase transition at a temperature of 385.80 K. During the subsequent cooling cycle, an exothermic peak at 384.52 K was observed, with a small thermal hysteresis of approximately 1.3 K. The enthalpy change, estimated from the DSC curve, remained consistent at 0.9–1.2 kJ mol^−1^ for both cooling and heating processes. Notably, after subjecting the crystals to 60 heating and cooling cycles, the transition temperature and enthalpy remained unchanged (Fig. 1B and Table S1†), indicating a high level of reversibility of this phase transition. We will henceforth refer to the two phases as the low-temperature (LT) form and the high-temperature (HT) form.

Subsequently, PCV crystals were subjected to single-crystal X-ray diffraction above (400 K) and below (380 K) the transition point. The obtained crystallographic parameters are summarized in Table S2.† It reveals that the LT form (CCDC 2313630) belongs to the *P*2_1_/*c* space group with only one PCV molecule in the asymmetric unit. Within the LT form, PCV molecules form an infinite hydrogen-bonded tape along the *b*-axis, which is further connected through O–H⋯N and N–H⋯O bonds, resulting in a 2D layered structure (Fig. S2A†). These layers further stack along the [101̄] direction and interact with each other by the weak hydrogen bond as C=O⋯H–O (Fig. 1C). The centroid–centroid distance of purine rings within each adjacent layer is equal (4.52 Å). The HT form exhibits a similar layered structure to the LT form. In contrast, the HT form (CCDC 2313632) crystallizes in a triclinic system (*P*1̄ space group) and contains two PCV molecules in the asymmetric unit, resulting in the centroid–centroid distance changes from the equal 4.52 Å to 4.50 Å and 4.90 Å, respectively (Fig. 1C). While the *b*-axis orientation in the HT form is consistent with the LT form, the orientations of the *a* and *c* axes in the HT form correspond approximately to the orientation of the *c*-axis and the opposite orientation of the *a*-axis in the LT form, respectively.

**Fig. 2:**
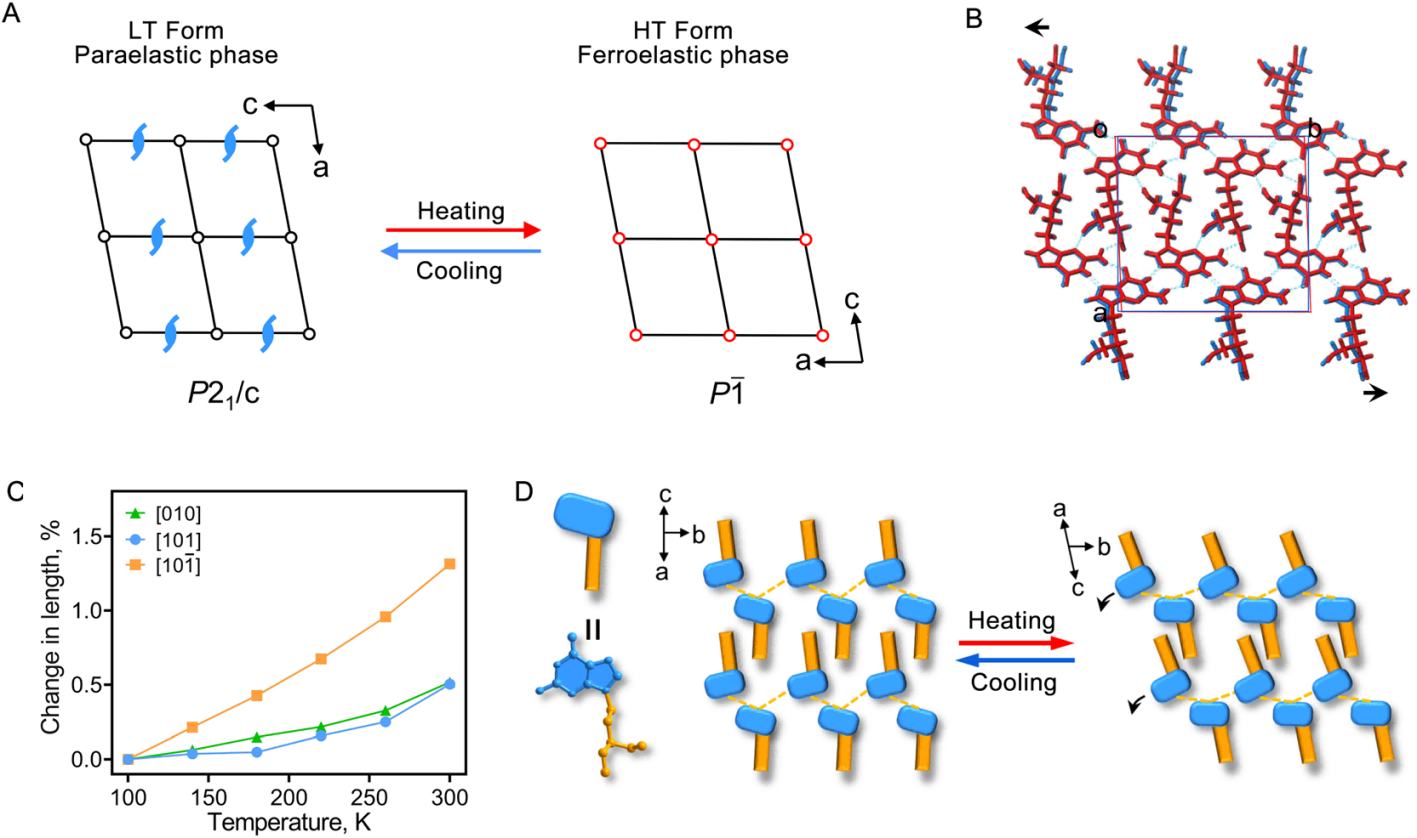
Molecular mechanism for inverse temperature symmetry breaking of the PCV crystals. (A) Transformation of the space group for the PCV crystals from the LT form (paraelastic phase) to the HT form (ferroelastic phase). (B) Superposition of the molecular packing structures of the LT (in blue) and HT (in red) forms viewed along the *c*-axis_LT_. Black arrows indicate the relative displacements of PCV molecules during the LT to HT form transition. (C) Variation of the principal axes concerning temperature for the LT form. (D) Schematic illustration of the molecular mechanism of the ITSB. Black arrows indicate the tilt of PCV molecules during the LT to HT form transition.

The lattice symmetry generally increases during a phase transition from the LT to the HT form, and such a type of structural phase transition is called a symmetry-breaking phase transition.^15^ However, in the case of PCV crystals, we observed that the transition from the *P*2_1_/*c* space group (the LT form) to the *P*1̄ space group (the HT form) leads to a reduction in the number of symmetry elements from 4 (*E*, *C*_2_, *i*, and *σ*_h_) to 2 (*E* and *i*) (Fig. 2A and S3†), indicating an unusually inverse temperature symmetry breaking (ITSB). To understand this anomalous ITSB in PCV crystals, unit cell parameters were collected before the LT to HT form transition (Table S3†). The results revealed a relatively weak and isotropic thermal expansion within the molecular layer ((101̄)), not exceeding 0.5% along the [101] and [010] directions, with an approximately 1.3% elongation in the molecular layer stacking direction ([101̄] direction), indicating a stronger intermolecular interaction within the molecular layer (Fig. 2C, S2 and S4†). Notably, the hydrogen-bonded PCV molecules within the layered structure slightly tilt with increasing temperature, causing a displacement of molecules along the *b*-axis during the LT to HT phase transition (Fig. 2B and D). This process leads to the loss of symmetry elements, such as the 2-fold screw axis and *c*-glide plane, in the HT form.

**Fig. 3:**
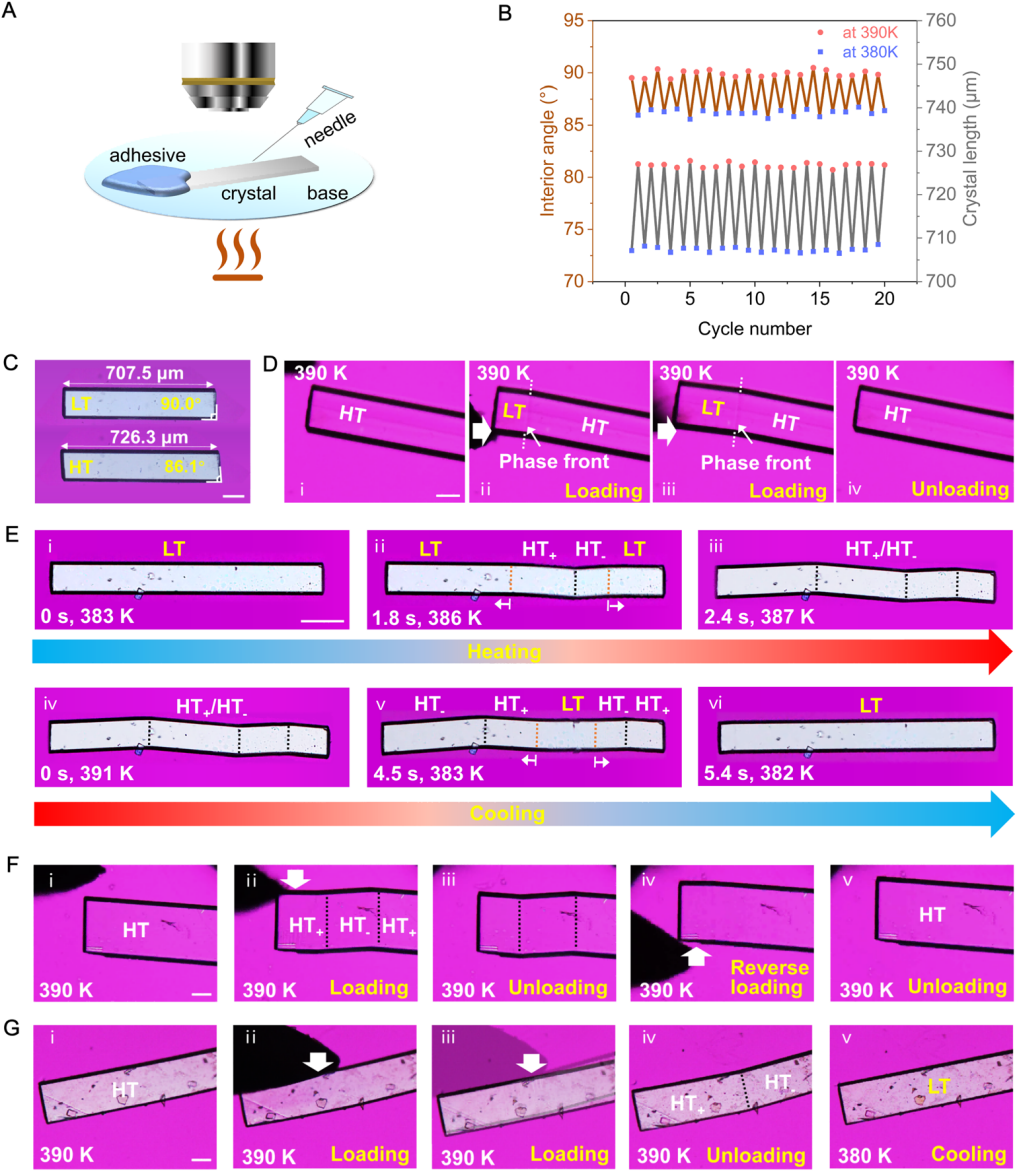
Thermoelasticity, superelasticity, ferroelasticity, and shape memory effects in the PCV crystals. (A) Experimental setup for shear mechanics. (B) Changes in crystal length and interior angle of a single crystal during 20 heating–cooling cycles. (C) The thermoelastic transition of a PCV crystal (Mov. S1†). (D) The superelastic transition of a PCV crystal (Mov. S7†). (E) Temperature evolution of the ferroelastic domain structures in the (100)_LT_ ((001)_HT_) plane and the reversible shape deformation during the heating and cooling process (Mov. S2†). (F) Reversible twinning deformation of PCV HT form prepared by compression along the [010] direction. ii, forward direction, iv, reverse direction (Mov. S4†). (G) Shape-recovery *via* cooling the mechanical twinned crystal with the generation of LT form through the HT to LT phase transition (Mov. S5†). All scale bars are equal to 100 μm.

We employed polarized optical microscopy equipped with a hot stage (Fig. 3A) to investigate the single-crystal-to-single-crystal (SCSC) phase transition in PCV. Face indexing results revealed that the basal face exposed to the hot stage is parallel to the (100) plane of the LT form and the (001) plane of the HT form, respectively (Fig. S5†). Under the optical microscope, the LT and HT forms could be distinguished by measuring and comparing the interior angles of PCV single crystals (Fig. 3C). Notably, PCV crystals of both forms are exceptionally robust during the thermal-induced phase transition, retaining their single-crystal properties even after twenty cycles (Fig. 3B and C, Mov. S1†).

**Fig. 4:**
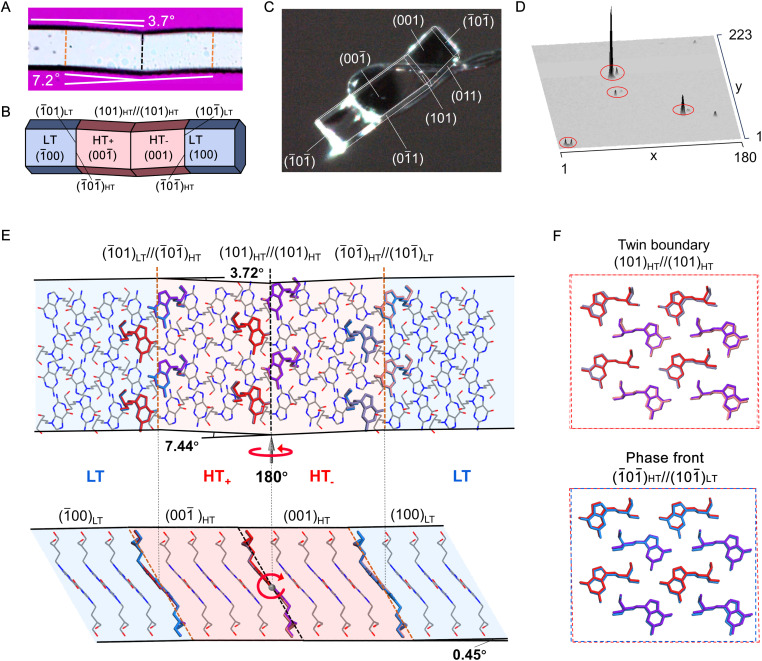
Analysis of twinning in the PCV crystals. (A) A photo of a deformed PCV crystal upon heating. Orange dash lines represent the LT to HT phase front, and black dash lines represent twin boundaries in the HT form. (B) A schematic representation of the deformed crystal with face indices. (C) Face indexing of a twinned PCV crystal at 400 K. (D) 3D intensity profile of diffraction patterns for a twinned PCV crystal at 400 K. The doubled spots were observed (marked by red cycles). (E) Molecular correspondence at the interface for LT//HT and HT_+_//HT_−_. Molecules at the interface are coloured in blue in LT form, red (Z1) and purple (Z2) in HT_+_ form, and pink (Z1) and light purple (Z2) in the HT_−_ form, respectively. (F) Molecular positions on the twinning boundary and phase front.

According to Aizu's notation, the phase transition of PCV can be classified into the 2/mF1̄ species and has a ferroelastic character.^18,44^ The evolution of twinning deformation in PCV HT form when exposed to heat is shown in Fig. 3E. When heated to 387 K, the straight rod-shaped crystal bent into four recognizable domains, with domain walls intersected at angles of 7.2° (Fig. 4A). Upon cooling the crystal back to 382 K, it regained its straight shape, demonstrating thermal shape recovery behaviour (Mov. S2†). According to the crystal indices in the bent shape (Fig. 4C), the deformation occurs through a 180° rotational twining around the [010] direction, resulting in (101)_HT_+__//(101)_HT_−__ as the twinning interface (Fig. 4E). The predicted bending angle between HT_+_ and HT_−_ is 7.44°, which agrees well with the measured bending angle (7.2°) from the optical microscope (Fig. 4A). The doubled spots in the 3D intensity profile of the diffraction patterns further confirmed the twinning deformation of the PCV HT forms (Fig. 4D). Meanwhile, during the heating and cooling processes, we observed a phase boundary line (orange dotted line) in Fig. 3E(ii and v) sweeping across the crystal sample at a rate of 0.1–10.0 mm s^−1^, which resulted in crystal bending with an angle of 3.7° (Fig. 4A). This movable phase boundary was more pronounced in plate-like crystals (Fig. S6 and Mov. S3†). According to the crystallographic analysis, the phase boundaries are supposed to be (1̄01)_LT_//(1̄01̄)_HT_ and (1̄01̄)_HT_//(101̄)_LT_ interfaces because the calculated bending angle between (1̄01)_LT_//(1̄01̄)_HT_ is 3.72° (Fig. 4B).

Under shearing stress in the [010] direction, the PCV HT form underwent twinning deformation (Fig. 3F and Mov. S4†), creating a daughter domain (HT_−_) with a constant bending angle of approximately 7° (Fig. S7†). The bent crystal was subsequently restored to its original straight shape by shearing in the opposite direction (Fig. 3F(iv and v)). Moreover, the mechanically twinned crystal can also regain its straight shape by cooling (Fig. 3G and Mov. S5†). Upon loading in the [010] or [100] direction, superelastic deformation of the HT form was also observed, with a bending angle of approximately 3.7°, indicating the generation of the LT form (Fig. 3D and S8, Mov. S6 and S7†). Notably, despite its mechanical flexibility at high temperatures, the LT form of PCV remained rigid and brittle (Fig. S9 and Mov. S8†).

To understand the structural basis for the ferro- and superelasticity of the PCV HT forms, we analysed the crystal structure at the deformation interfaces. In the HT form, there are two crystallographically independent molecules, Z1 and Z2, exhibiting highly similar molecular conformations (Fig. S10 and S11†). During twinning deformation, Z1(HT_+_) and Z2 (HT_+_) at the interface covert into Z2 (HT_−_) and Z1 (HT_−_), respectively (Fig. 4E and F). Concurrently, the molecules undergo slight displacement along the *b* and *c* axes_LT_ (Fig. S12†). In the case of superelastic deformation, two crystallographically independent molecules in an HT domain convert to those shown in an LT domain. The estimated molecular movements at these interfaces resemble those observed in twinning deformation but involve relatively large changes in molecular positions (Fig. 4F and S12†).

**Fig. 5:**
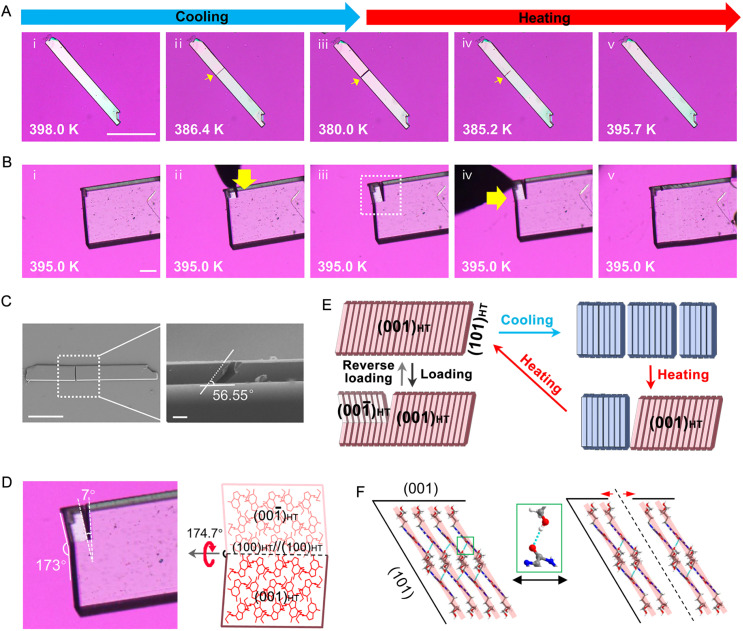
Self-healing behaviours of the PCV crystals. (A) A crystal cracked during the first heating–cooling cycle over the phase transition HT to LT and healed during the second heating process (Mov. S10†). The white arrow shows the region where the crack occurred and disappeared. Scale bar: 100 μm. (B) The cracking and healing process of a crystal of HT form under mechanical stress (Mov. S12†). Scale bar: 100 μm. (C) SEM image of a cracked crystal at room temperature. A vertical view (scale bar: 100 μm) and an enlarged side view (scale bar: 10 μm). (D) An enlarged view and related molecular mechanism of the dotted box in B(iii). (E) The postulated mechanism of cracking to self-healing. (F) During the cracking-to-self-healing, the interlayer hydrogen bonds (C=O⋯H–O) rapidly break and regenerate.

Surprisingly, some PCV crystals exhibited a remarkable self-healing behaviour during the thermally-induced phase transition. As shown in Fig. 5A, in seldom cases, cracks appeared on the basal face during the HT to LT transition, yet upon heating the crystal above the LT to HT transition temperature, the cracks gradually disappeared (Fig. S13 and Mov. S9†), with some crystals fully recovering their original state (Mov. S10 and S11†). These completely healed crystals exhibited diffraction patterns identical to the HT form with excellent shape and intensities, suggesting that the crystal has recovered its single crystalline nature (Fig. S14†). Scanning electron microscope (SEM) images revealed that the cracks typically tilted along the long axis at approximately 60° (Fig. 5C), suggesting PCV crystals tend to split along the direction parallel to the (101)_HT_ ((101̄)_LT_) plane (Fig. S15†). As mentioned above, the (101)_HT_ ((101̄)_LT_) plane is a typical slip plane in both PCV forms due to the layered structure and the weakest interlayer interaction (Fig. S16 and S17†),^45^ where slippage and cleavage easily occur when exposed to stresses. During the HT to LT phase transition (400 K to 380 K), the PCV crystals underwent significant anisotropic contraction, experiencing a 1.76% contraction along the long axis, in contrast to changes along the other two axes, each less than 0.5%. These changes generated greater stress along the long axis, ultimately leading to crack formation along the slippage plane ((101)_HT_ ((101̄)_LT_)) after surpassing critical conditions. We propose that as the cracked crystal undergoes the LT to HT phase transition upon heating, fragments tend to slide back, bridging the crack interfaces into proximity (Fig. 5E), and the rapid reformation of C=O⋯H–O hydrogen bonds across the cracked interface subsequently facilitates the crack healing (Fig. 5F).

The HT form of PCV crystals displayed a remarkable recovery even after mechanical cracking, a phenomenon rarely observed in crystalline materials (Fig. 5B, Mov. S12 and S13†). In this scenario, the crystal initially fractured into bifurcated branches along its *b*-axis under local pressure from a needle. Measurement of the inner and intersected angles of the two fragments indicated that the cracking resulted from a twinning deformation along the (100)_HT_ plane, resulting in the angle of the crack not exceeding 7° (Fig. 5D). Subsequently, gentle pressure along the [001]_HT_ direction reversed the twinning deformation and effectively restored the crystal. According to SEM images, the crystals also cleaved along the (101)_HT_ plane under mechanical stress (Fig. S18†). It is thus reasonable to posit that these crystals share a similar self-healing mechanism akin to thermally induced cracked crystals which are facilitated by the reformation of hydrogen bonds with mechanical force bringing the cracked interface back together.^46–49^

## Conclusion

In summary, we present the discovery of a mechanical responsive and adaptive molecular crystal composed of an antivirus drug PCV. This crystal exhibits an unconventional ferroelastic phase transition through inverse temperature symmetry breaking, triggered by cooperative molecular displacement. Both polymorphs feature a typical layered crystal structure. When transforming into a ferroelastic phase at elevated temperatures, PCV crystals demonstrate a diverse range of memory and restorative effects, governed by molecular motion within the molecular layer. Furthermore, these ferroelastic crystals display a remarkable self-healing capability in response to both thermal and mechanical-induced cracks. We propose that the smooth layer structure and rapid reformation of the dynamic interlayer hydrogen bonds are responsible for these self-healing behaviours. Our findings contribute to the fundamental understanding of cooperative transitions in molecular crystals and provide valuable insights for designing new types of flexible organic crystalline materials with controllability, reversibility, and durability.

## Data availability

The data that support the findings of this study are available from the corresponding author upon reasonable request.

## Author contributions

J. T. M. and Y. S. contributed equally to this work. J. T. M., Y. S and T. C. conceived the study, analysed the experiment data, wrote and edited the manuscript. Y. S. and T. C. obtained the fund. J. T. M. and H. Z. performed the experiment. Y. S. carried out the crystal structure analysis. All the authors discussed the results and approved the final version of the manuscript.

## Conflicts of interest

There are no conflicts to declare.

## Supplementary Material

SC-015-D3SC06800E-s001

SC-015-D3SC06800E-s002

SC-015-D3SC06800E-s003

SC-015-D3SC06800E-s004

SC-015-D3SC06800E-s005

SC-015-D3SC06800E-s006

SC-015-D3SC06800E-s007

SC-015-D3SC06800E-s008

SC-015-D3SC06800E-s009

SC-015-D3SC06800E-s010

SC-015-D3SC06800E-s011

SC-015-D3SC06800E-s012

SC-015-D3SC06800E-s013

SC-015-D3SC06800E-s014

SC-015-D3SC06800E-s015
