# Supplementary material for: Shape memory and self-healing in a molecular crystal with inverse temperature symmetry breaking
Source: Chem Sci. 2024 Mar 8;15(15):5738–45. doi: 10.1039/d3sc06800e (PMC11023024; doi:10.1039/d3sc06800e)
Supplement: SC-015-D3SC06800E-s001 [file SC-015-D3SC06800E-s001.pdf]

Electronic Supplementary Material (ESI) for Chemical Science.
This journal is © The Royal Society of Chemistry 2024

Shape Memory and Self-Healing in a Molecular Crystal with Inverse Temperature Symmetry Breaking†

Jiantao Meng,‡[a] Yuan Su,‡[b], Hang Zhu,[a] and Ting Cai*[a, b]

Table of Contents

Experimental Procedures 3

Reagents and Crystal Growth 3

Characterizations 3

Results and Discussion 4

Figure S1. Morphology of the PCV crystals. 4

Figure S2. Molecular packing of the LT and HT forms. 4

Figure S3. Molecular mechanism for inverse temperature symmetry breaking of the PCV crystals. 4

Figure S4. Type and distribution of hydrogen bonding interactions in the LT form. 4

Figure S5. Face indexing of a PCV crystal. 5

Figure S6. The reversible phase transition between LT form and HT form. 5

Figure S7. A PLM image of a mechanical twinned PCV crystal. 5

Figure S8. Multi-directional superelasticity of a PCV crystal. 6

Figure S9. A PCV crystal fractured at room temperature under loading. 6

Figure S10. Conformation of independent molecule in the LT and HT forms. 6

Figure S11. The angle between the centroid connection and the bottom edge in PCV molecules. 6

Figure S12. Molecular position in the LT and HT forms. 7

Figure S13. The morphology of an imperfect healed PCV crystal. 7

Figure S14. Diffraction patterns of a PCV crystal under the cracked and healed state. 7

Figure S15. Measurement of the angle between the simulated basal surface and the cleavage surface. 7

Figure S16. Intermolecular interaction energies of the LT and HT forms. 8

Figure S17. Energy framework of the LT and HT forms. 8

Figure S18. A SEM image of the cleavage surface in the PCV crystal. 8

Table S1. Phase transition temperatures and enthalpy values of the PCV crystals. 9

Table S2. Crystallographic data of the PCV polymorphs. 9

Table S3. Partial crystallographic data of the LT form in different temperatures. 10

Table S4. Unit cell parameters of a twinned PCV crystal at 400 K. 10

References 10

Movie S1. The thermoelastic transformation of a PCV crystal in different cycles.

Movie S2. The reversible shape deformation of the PCV crystals during the heating-cooling process.

Movie S3. A phase boundary line sweeping across a PCV crystal during the reversible phase transition.

Movie S4. The ferroelastic transformation of a PCV crystal.

Movie S5. The shape memory effect of a PCV crystal.

Movies S6-S7. The superelastic transformation of a PCV crystal.

Movie S8. A PCV crystal fractured at room temperature under loading.

Movie S9. An imperfect self-healing effect of a cracked PCV crystal during the heating process.

Movies S10-S11. Cracking and healing of the PCV crystals during the heating and cooling process.

Movies S12-S13. The cracking-healing process of the PCV HT forms under mechanical stimulation.

Experimental Procedures

Reagents and Crystal Growth

Penciclovir PCV (98%) was purchased from Aladdin Reagent Co., Ltd. (Shanghai, China). Analytical-grade solvents were used for the crystallization experiments. All materials were used as received.

The excessive powder was added to ethanol-H_2O mixed solvent, and the system was heated to 310 K and stirred for 1 hour. After filtered, the filtrate was slowly evaporated at room temperature. Plate-like or rod-like colorless single crystals were obtained after 3 days.

Characterization

DSC analysis was conducted in standard aluminum pans using a TA Instrument Q2000 unit under 50 mL min^{-1} nitrogen gas purge. Accurately weighed samples (2-8 mg) were loaded in sealed aluminum pans and analyzed from 300 K to 420 K at a heating rate of 10 K min^{-1}.

Thermoelasticity, shape memory effects, and self-healing were investigated within a temperature range of 370 K to 410 K. Superelasticity, ferroelasticity, and healing under mechanical stress were tested at 390 K, before inducing crystal deformation with mechanical stress, one end of the crystal was fixed in advance on the glass sheet. All the macroscopic responses were visually recorded using a hot stage assisted microscopy (Olympus BX53), and heating/cooling cycles were monitored by Linkam THMS 600.

The scanning electron microscopy (SEM) characterization was carried out with a TESCAN MIRA S6123 electron microscope with a primary electron energy of 3 keV. The SEM images were collected with a TESCAN Essence Version 1.1.4.0.

Fluorescent images of the PCV crystal were recorded by a Nikon inverted fluorescence microscope (ECLIPSE Ts2R, Nikon, Tokyo, Japan) at the UV-1A channel. The images were analyzed using fluorescent software (NIS-Elements F 5.21.00, Nikon).

Single-crystal X-ray diffraction data were collected using a Bruker D8 Venture X-ray diffractometer (Mo Kα, 0.71073 Å). The single crystal structure was analyzed using ShelXS-2014/7 (Sheldrick, 2014). Absorption correction was performed using the SADABS program and structural correction was performed using the full matrix least square method. Face indexing and diffraction patterns analysis were carried out using the Rigaku HyPix (Cu Kα, 1.54184 Å) and CrysAlis^{Pro} 1.171.42.89a (Rigaku OD, 2023). Unit cell parameters were collected within the temperature range of 100 to 300 K, by gradual heating of the crystal at intervals of 40 K with the help of a cryojet.

Crystallographic data of two forms of PCV crystal have been deposited at the Cambridge Crystallographic Data Centre (CCDC, www.ccdc.cam.ac.uk/data_request/cif) under deposition numbers CCDC 2313630 (LT form) and 2313632 (HT form).

Results and Discussion

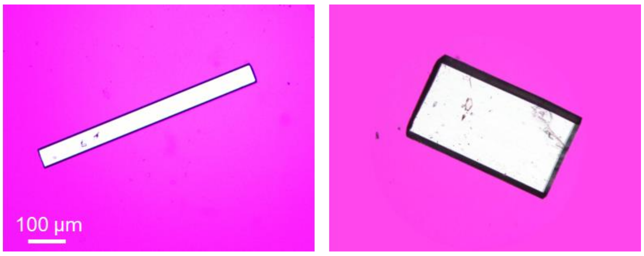

Figure S1. Rod-like and plate-like morphology of PCV single crystals cultivated from a mixed alcohol solution.

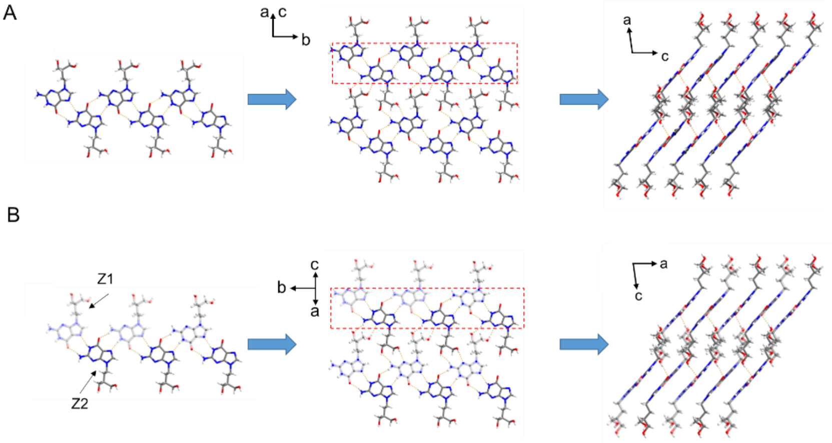

Figure S2. Molecular packing of the LT (A) and HT (B) forms. In both forms, the PCV molecules form an infinite one-dimensional (1D) hydrogen-bonded tape extending along the *b*-axis. The parallel tapes connect with other tapes via O-H…N and N-H…O hydrogen-bonding interactions, forming a corrugate 2D layer structure. The molecular layers stack further along the [10$\bar{1}$] and [101] direction in the LT and HT forms, respectively.

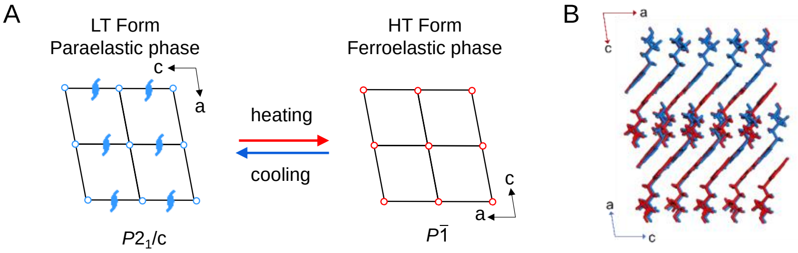

Figure S3. Molecular mechanism for inverse temperature symmetry breaking of the PCV crystals. (A) Transformation of the space group for the PCV crystals from the LT form (paraelastic phase) to the HT form (ferroelastic phase). (B) Superposition of the molecular packing structures of the LT (in blue) and HT (in red) forms viewed along the *b*-axis, respectively.

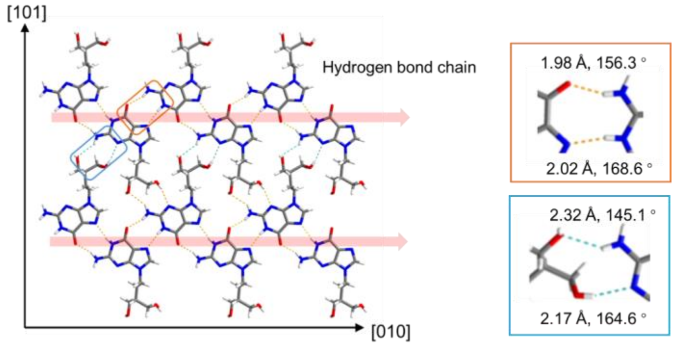

Figure S4. Type and distribution of hydrogen bonding interactions in (10$\bar{1}$) plane of the LT form. Changes along the [010] and [101] directions do not exceed 0.5% within the temperature range of 100 to 300 K.

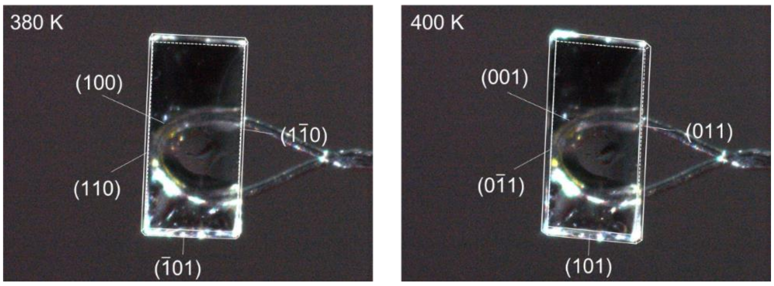

Figure S5. Face indexing of a PCV crystal at 380 and 400 K. The major crystal face is paralleled to the (100) and (001) plane of the LT and HT forms, respectively.

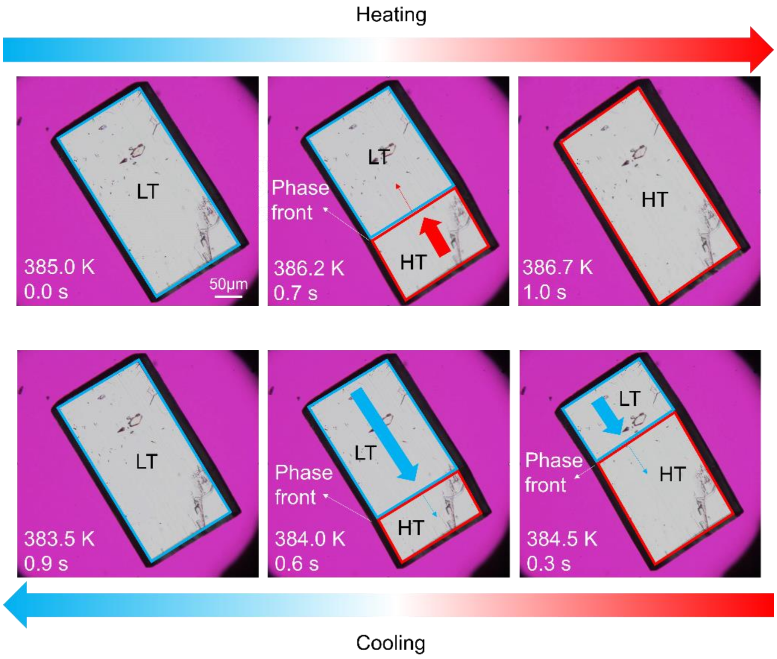

Figure S6. The reversible phase transition between PCV LT form and HT form. The position of the phase boundary is indicated with white arrows. The phase boundary line swept across the crystal sample at a rate of 0.1~10.0 mm/s.

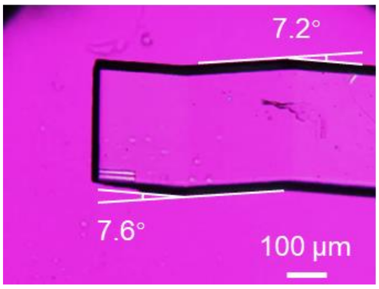

Figure S7. A PLM image of a mechanical twinned PCV crystal. Under shearing stress in the [010] direction, the PCV HT form underwent twinning deformation with a constant bending angle of approximately 7°.

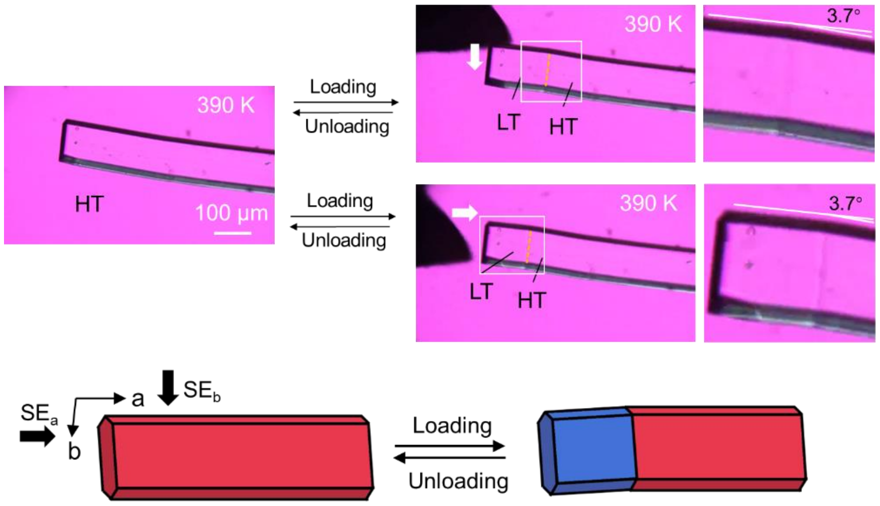

Figure S8. Multi-directional superelasticity (SE) of a PCV crystal based on the HT to LT phase transition. The directions in $[010]_{HT}$ and $[100]_{HT}$ in PCV crystal can be effective axes for shear-induced superelastic transition.

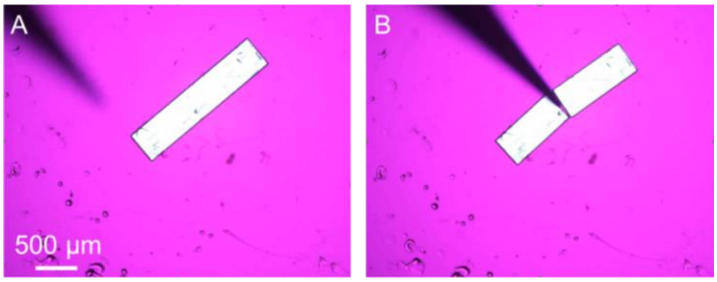

Figure S9. A PCV crystal fractured at room temperature under loading. (A) Initial state. (B) Cracked state.

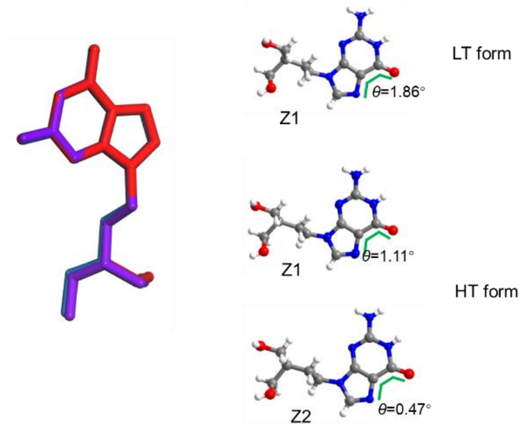

Figure S10. Conformation of independent molecule in the LT (blue) and HT (Z1: red and Z2: purple) forms.

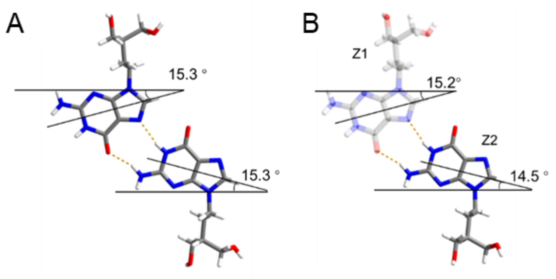

Figure S11. The angle between the centroid connection and the bottom edge of the independent molecule in the LT (A) and HT (B) forms. Two crystallographically independent molecules in HT form (Z1 and Z2) exhibit highly similar molecular conformations.

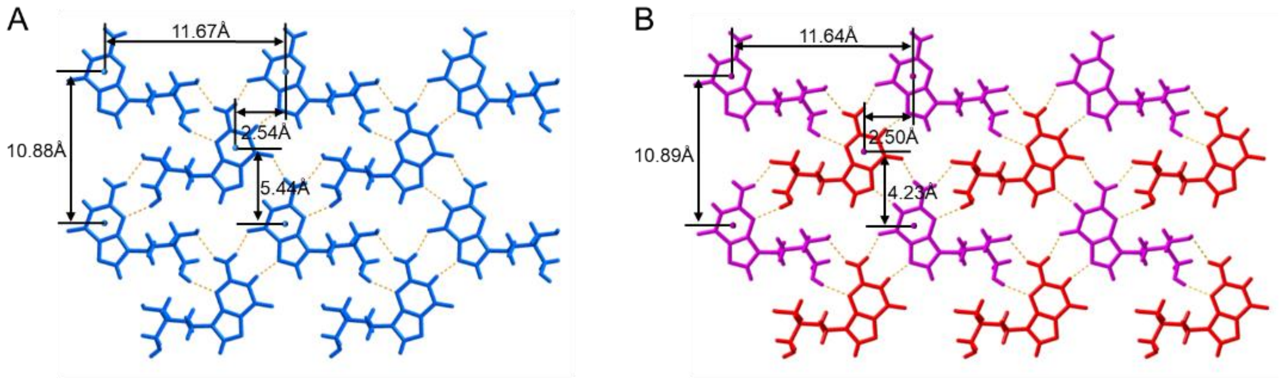

Figure S12. Molecular position in the LT (A) and HT (B) forms. The distances between molecules changed nonuniformly during the phase transition.

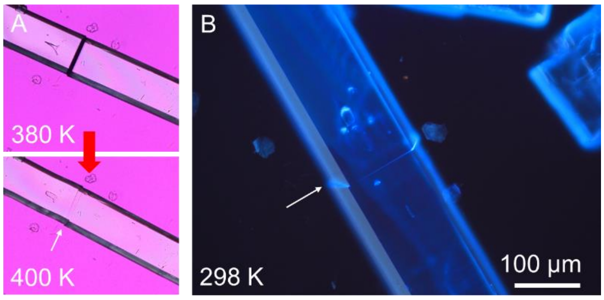

Figure S13. The morphology of an imperfect healed PCV crystal. (A) Polarized light microscopy images of an imperfect healing process. (B) A Fluorescence image of the imperfect healed PCV crystal at room temperature. The white arrow shows the region partially healed.

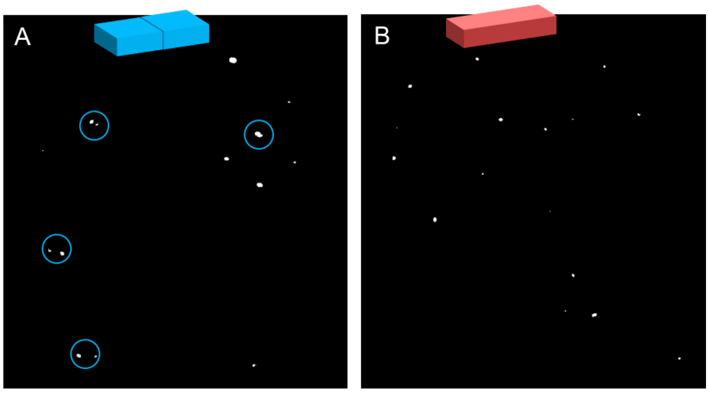

Figure S14. Diffraction patterns of a PCV crystal under the cracked (A) and healed (B) state. The doubled or split spots at 380 K represent the crystal is in a cracked state, clear diffraction patterns demonstrate the perfect healing of crystals after phase transition (400 K).

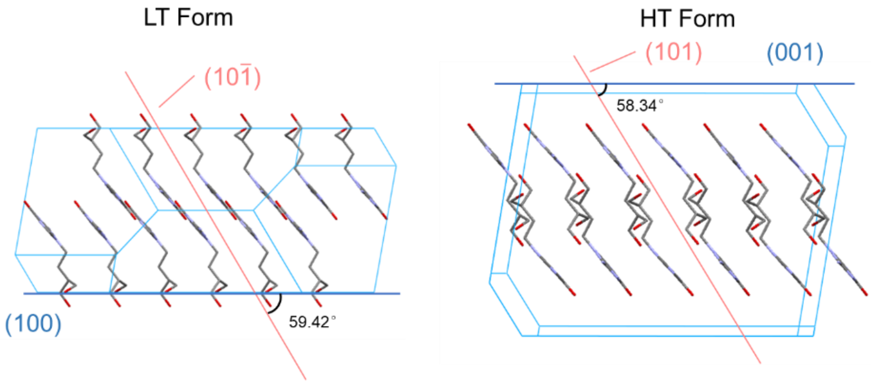

Figure S15. Measurement of the angle between the simulated basal surface and cleavage surface of the PCV crystals. The basal surfaces for the LT and HT forms are parallel to (100) and (001), respectively. The cleavage surfaces for the LT and HT forms are parallel to (10$\bar{1}$) and (101), respectively.

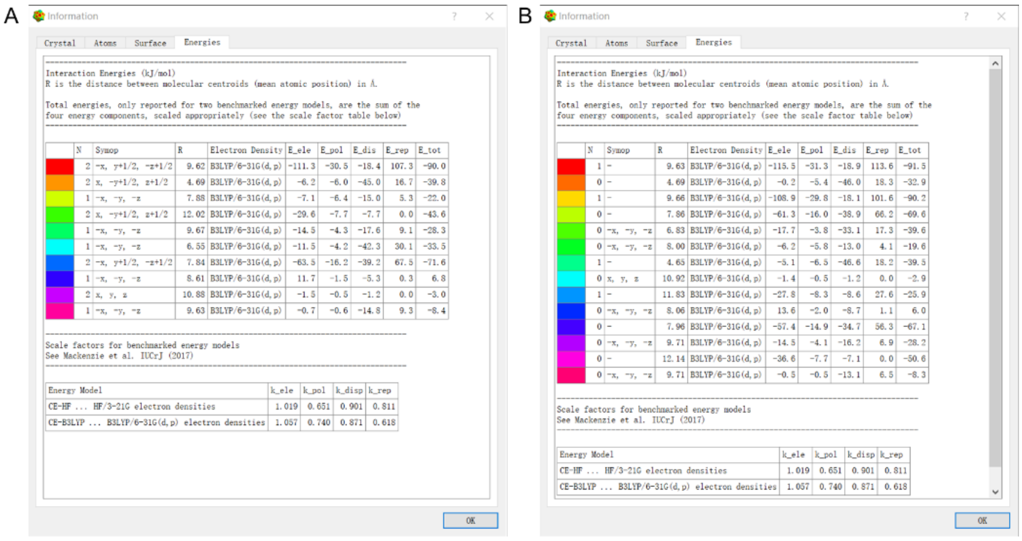
A

Information

Crystal | Atoms | Surface | Energies

Interaction Energies (kJ/mol)
R is the distance between molecular centroids (mean atomic position) in Å.

Total energies, only reported for two benchmarked energy models, are the sum of the four energy components, scaled appropriately (see the scale factor table below)

	N	Symop	R	Electron Density	E_ele	E_pol	E_dis	E_rep	E_tot
	2	-x, y+1/2, -z+1/2	9.62	B3LYP/6-31G(d,p)	-111.3	-30.5	-18.4	107.3	-90.0
	2	x, -y+1/2, z+1/2	4.69	B3LYP/6-31G(d,p)	-6.2	-6.0	-45.0	16.7	-39.8
	1	-x, -y, -z	7.88	B3LYP/6-31G(d,p)	-7.1	-6.4	-15.0	5.3	-22.0
	2	x, -y+1/2, z+1/2	12.02	B3LYP/6-31G(d,p)	-29.6	-7.7	-7.7	0.0	-43.6
	1	-x, -y, -z	9.67	B3LYP/6-31G(d,p)	-14.5	-4.3	-17.6	9.1	-28.3
	1	-x, -y, -z	6.55	B3LYP/6-31G(d,p)	-11.5	-4.2	-42.3	30.1	-33.5
	2	-x, y+1/2, -z+1/2	7.84	B3LYP/6-31G(d,p)	-63.5	-16.2	-39.2	67.5	-71.6
	1	-x, -y, -z	8.61	B3LYP/6-31G(d,p)	11.7	-1.5	-5.3	0.3	6.8
	2	x, y, z	10.88	B3LYP/6-31G(d,p)	-1.5	-0.5	-1.2	0.0	-3.0
	1	-x, -y, -z	9.63	B3LYP/6-31G(d,p)	-0.7	-0.6	-14.8	9.3	-8.4

Scale factors for benchmarked energy models
See Mackenzie et al. IUCrJ (2017)

Energy Model	k_ele	k_pol	k_disp	k_rep
CE-HF ... HF/3-21G electron densities	1.019	0.651	0.901	0.811
CE-B3LYP ... B3LYP/6-31G(d,p) electron densities	1.057	0.740	0.871	0.618

OK

B

Information

Crystal | Atoms | Surface | Energies

Interaction Energies (kJ/mol)
R is the distance between molecular centroids (mean atomic position) in Å.

Total energies, only reported for two benchmarked energy models, are the sum of the four energy components, scaled appropriately (see the scale factor table below)

	N	Symop	R	Electron Density	E_ele	E_pol	E_dis	E_rep	E_tot
	1	-	9.63	B3LYP/6-31G(d,p)	-115.5	-31.3	-18.9	113.6	-91.5
	0	-	4.69	B3LYP/6-31G(d,p)	-0.2	-5.4	-46.0	18.3	-32.9
	1	-	9.66	B3LYP/6-31G(d,p)	-108.9	-29.8	-18.1	101.6	-90.2
	0	-	7.86	B3LYP/6-31G(d,p)	-61.3	-16.0	-38.9	66.2	-69.6
	0	-x, -y, -z	6.83	B3LYP/6-31G(d,p)	-17.7	-3.8	-33.1	17.3	-39.6
	0	-x, -y, -z	8.00	B3LYP/6-31G(d,p)	-6.2	-5.8	-13.0	4.1	-19.6
	1	-	4.65	B3LYP/6-31G(d,p)	-5.1	-6.5	-46.6	18.2	-39.5
	0	x, y, z	10.92	B3LYP/6-31G(d,p)	-1.4	-0.5	-1.2	0.0	-2.9
	1	-	11.83	B3LYP/6-31G(d,p)	-27.8	-8.3	-8.6	27.6	-25.9
	0	-x, -y, -z	8.06	B3LYP/6-31G(d,p)	13.6	-2.0	-8.7	1.1	6.0
	0	-	7.96	B3LYP/6-31G(d,p)	-57.4	-14.9	-34.7	56.3	-67.1
	0	-x, -y, -z	9.71	B3LYP/6-31G(d,p)	-14.5	-4.1	-16.2	6.9	-28.2
	0	-	12.14	B3LYP/6-31G(d,p)	-36.6	-7.7	-7.1	0.0	-50.6
	0	-x, -y, -z	9.71	B3LYP/6-31G(d,p)	-0.5	-0.5	-13.1	6.5	-8.3

Scale factors for benchmarked energy models
See Mackenzie et al. IUCrJ (2017)

Energy Model	k_ele	k_pol	k_disp	k_rep
CE-HF ... HF/3-21G electron densities	1.019	0.651	0.901	0.811
CE-B3LYP ... B3LYP/6-31G(d,p) electron densities	1.057	0.740	0.871	0.618

OK

Figure S16. Intermolecular interaction energies of the LT (A) and HT (B) forms calculated using Crystal Explorer 21 (B3LYP/6-31G(d,p)).[1]

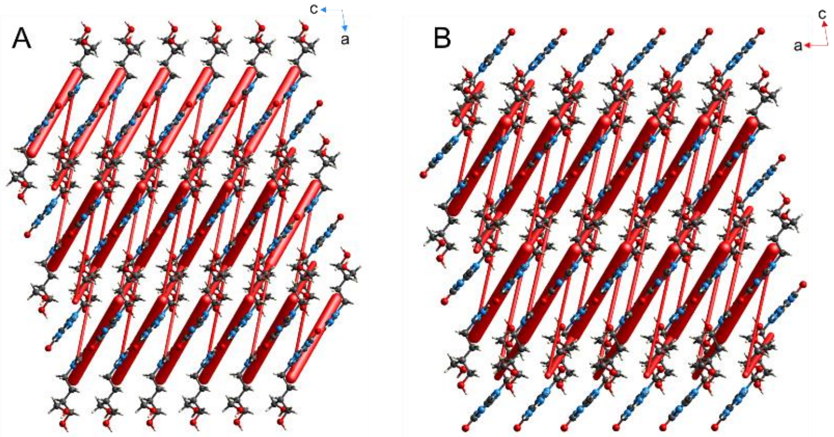

Figure S17. Energy framework of the LT (A) and HT (B) forms. The thickness and direction of the rod-shaped structure represent the direction and magnitude of strong interactions within the crystal, respectively. The rod size in the energy frameworks is set to 50, and the cut-off energy value is 20 kJ·mol^{-1}.

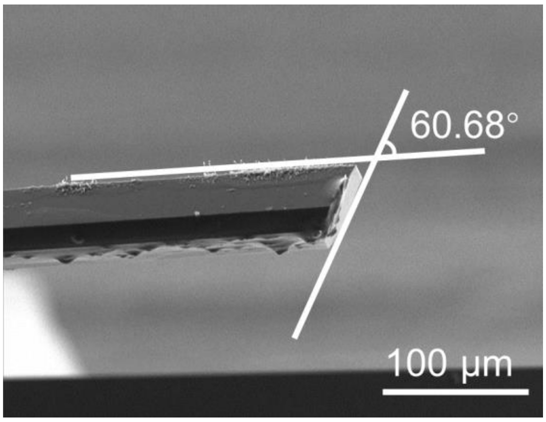

Figure S18. A SEM image of the cleavage surface in the PCV crystal. The HT form of PCV crystal fractured completely under mechanical stress. The cleavage surface is parallel to the $(10\bar{1})_{LT}$ ($(101)_{HT}$) plane.

Table S1. Phase transition temperatures and enthalpy values of the PCV crystals under different heating-cooling cycles.

Cycle	T_t-heating, K	ΔH, kJ·mol^{-1}	T_t -cooling, K	ΔH, kJ·mol^{-1}
1	385.82	0.99	384.53	1.13
10	385.69	1.14	384.55	1.20
30	385.92	0.96	384.43	1.09
60	385.79	1.13	384.56	1.17

Table S2. Crystallographic data of the PCV polymorphs.

	PCV LT form	PCV HT form
CCDC number	2313630	2313632
Empirical formula	$C_{10}H_{15}N_5O_3$	$C_{10}H_{15}N_5O_3$
Formula weight	253.27	253.27
Temperature/K	380	400
Crystal system	monoclinic	triclinic
Space group	$P2_1/c$	$P\bar{1}$
a/Å	11.8542(16)	9.093(4)
b/Å	10.8829(13)	10.918(4)
c/Å	8.9354(12)	11.805(4)
α/°	90	87.335(12)
β/°	99.888(5)	80.598(13)
γ/°	90	86.279(15)
Volume/Å^3	1135.6(3)	1153.1(8)
Z/Z'	4/1	4/2
ρ_{calc} g/cm^3	1.481	1.459
μ/mm^{-1}	0.113	0.111
F(000)	536.0	536.0
Crystal size/mm^3	0.28 × 0.15 × 0.12	0.28 × 0.15 × 0.12
Radiation	MoKα (λ = 0.71073)	MoKα (λ = 0.71073)
2Θ range for data collection/°	5.116 to 52.784	3.74 to 52.902
Index ranges	$-14 \le h \le 14$, $-13 \le k \le 13$, $-11 \le l \le 11$	$-11 \le h \le 11$, $-13 \le k \le 13$, $-14 \le l \le 14$
Reflections collected	8424	13021
Independent reflections	2312 [R_{int} =0.0599, R_{sigma} = 0.0601]	4689 [R_{int} =0.0557, R_{sigma} = 0.0701]
Data/restraints/parameters	2312/0/165	4689/7/329
Goodness-of-fit on F^2	1.031	1.074
Final R indexes [I>=2σ (I)]	R_1 = 0.0560, wR_2 = 0.1228	R_1 = 0.0729, wR_2 = 0.1664
Final R indexes [all data]	R_1 = 0.1107, wR_2 = 0.1556	R_1 = 0.1243, wR_2 = 0.1971
Largest diff. peak/hole / e Å^{-3}	0.24/-0.22	0.41/-0.23

Table S3. Partial crystallographic data of the LT form in different temperatures.

T, K	*a*/Å	*b*/Å	*c*/Å	*α*/°	*β*/°	*γ*/°	*V*/Å^3
100	11.7371	10.7863	8.7252	90	99.2810	90	1090.144
140	11.7474	10.7931	8.7456	90	99.3817	90	1094.034
180	11.7557	10.8024	8.7656	90	99.4966	90	1097.886
220	11.7712	10.8098	8.7911	90	99.5718	90	1103.035
260	11.7883	10.8216	8.8178	90	99.6793	90	1108.854
300	11.8209	10.8421	8.8506	90	99.7351	90	1117.989

Table S4. Unit cell parameters of a twinned PCV crystal at 400 K.

	a/Å	*b*/Å	*c*/Å	*α*/°	*β*/°	*γ*/°	*V*/Å^3	Ratio/%
Domain-1	9.12	10.93	11.78	87.32	80.45	86.44	1154.1	73.60
Domain-2	9.25	10.93	11.80	87.19	80.47	85.92	1172.8	26.40

References

1. P. R. Spackman, M. J. Turner, J. J. McKinnon, S. K. Wolff, D. J. Grimwood, D. Jayatilaka and M. A. Spackman, CrystalExplorer: a program for Hirshfeld surface analysis, visualization and quantitative analysis of molecular crystals, *J. Appl. Crystallogr.*, 2021, **54**, 1006-1011.
